# Farmers and Farming

**Published:** 1890-03

**Authors:** S. H. Preston


### FARMERS AND FARMING.

BY S. H. PRESTON.

The condition of the farmer has greatly improved during the last generation. A judicious manager can now carry on a farm five times as large as his grandfather's, with less outlay of hard-handed labor. This is owing to the wonderful improvement in agricultural implements and the invention of labor-saving machines.

But it is far different with the farmer's wife. Her labor, as compared with her grandmother's is greatly increased. The falling off in the health of farmers' children, so frequently remarked, may be ascribed to this cause—the over-worked condition of their mothers. It is a fact that the families of farmers now-a-days lack the hardihood of those of "ye olden time."

While inventions and improvements have been lightening the labor of the farmer himself, there has been no decrease in the domestic drudgeries of his wife. For instance : The business of butter making is as burdensome now as in the days of our grandmothers. But the wife has not much better means for making it than her grandmother had, with nearly the same unimproved and inconvenient implements of kitchen toil. She has not much better things to cook with, nor better things to cook. She has pretty much the same kind of kitchen, where she as well as the beef is roasted, and appurtenances accordingly.

The lives of many farmers' wives are wretched. They are slaves of hard and unrequited work. This is a shame. If husbandmen are not able to hire help, let them help their wives themselves. They should certainly see that their wives have the best of everything to work with. They should provide convenient cellars and cisterns, plenty of coal and wood, and something for washing better than a board and barrel.

We feel like saying a few friendly words to farmers. There is no reason why you should not have a happy home, if you are a rough-handed farmer, why you should not have consideration for your family. Make your cottage comfortable and cheerful. Fill it with joy and sunshine, and let your family grow about you like flowers. Plant trees, cultivate your gardens, beautify your yards, adorn your dwellings, and decorate your rooms. Put some plants along the front fence, and a pot of flowers by the doorway, even if there be no market value in them. They will certify to your culture and refinement, and put a blossom in your bosom that will be worth more than money.

Relinquish the idea that you will be ruined and forced to mortgage your farm if you happen to put the hog-pen back of the house and morning glories before it, if you do not get up at four o'clock in the morning and drudge till dark at irksome toil. Take leisure for reading, thinking, enjoyment and improving the minds and social natures of your wife and children. Keep up with the thoughts and discoveries of the day, and let love and happiness, as well as labor and lucre be the objects of life on the farm.

You can get happiness, even out of the ground. It is far easier, pleasanter and more profitable to carry on a farm the *right* way—to make a comfortable home and have agreeable surroundings, and feel that your family is contented and that you are a gentleman instead of a clod-hopper. Make your farming an occupation to be proud of. As a class, you are the only really independent people, and you might be the happiest and most envied. But you must make it something beside treadmill toil and continual care.

You want your boys to stay with you. Then don't make the farm so dreary and desolate and farming such a drudgery that they will start off to town the day they are twenty-one, to teach school, study theology, or keep store. Make it so attractive that they had rather remain at the happy old homestead than go out into the great boisterous world to become even boodle aldermen. If you wish to succeed as farmers, if you want to fill your fields with sunshine and all your years with joy, make home pleasant and make your wife and children comfortable and happy.

---

GREAT SCOTT! WHAT NEXT?—The Detroit *Journal* desires to receive, by postal card, the address of all living male and female descendants of Revolutionary officers and soldiers of 1776, and when possible, the name and state of the ancestor. A directory of warlike great-grandfathers would be a novel undertaking in a purely business way.

